# The importance of an exquisite approach: a case report of Perinatal loss

**DOI:** 10.1192/j.eurpsy.2025.1983

**Published:** 2025-08-26

**Authors:** A. Junquera, A. Gockel

**Affiliations:** 1 Psychiatry, HM Purta del Sur, Mostoles; 2 Psychiatry, Fuenlabrada University Hospital, Fuenlabrada, Spain

## Abstract

**Introduction:**

Perinatal loss is a painful process that we encounter on a regular basis in clinical practice but for which there is no adequate specific training. The treatment given to the mother will be decisive in mitigating the psychological consequences.

**Objectives:**

To raise awareness of the need to develop specific training plans in perinatal psychiatry. Such training should provide tools for an adequate psychotherapeutic approach, as well as updated protocols on the management of psychopharmacological medication.

**Methods:**

Descriptive report of a case of a women who came for consultation after having suffered an intrapartum fetal loss, based on the follow-up of the patient, with emphasis on the interventions performed during her stay in the hospital.

**Results:**

We report the case of a 39-week pregnant woman who suffered an intrapartum fetal loss. The psychiatry team was notified and the resident doctor in psychiatry, without prior assessment of the patient, prescribed lorazepam every 8 hours. The mother at that moment, as a result of the shock, refused to see the baby, which was immediately accepted by the staff without a suitable intervention to inform about the benefits of adequate perinatal care (spending time with the baby, use of swaddling clothes to avoid heat loss, memory box, avoid rushing). After being discharged from hospital, she only attended a gynecological check-up, with no relevant findings. Five months later, the patient attended a psychiatry consultation with a major depressive episode, reporting significant feelings of guilt for not having been able to say goodbye to her baby. She did not remember clearly anything of what happened and presented panic attacks when she had to go to the hospital, as well as the presence of self-harming ideas. At the same time, she expressed anger at the treatment received from the staff, to whom she attributes the fact that she was not able to spend time with the baby.

A recent study (Cassidy, 2023) shows how the use of sedatives has become normalized in Spanish hospitals in order to compensate for the deficits in training and resources offered by the health system.

Sedatives produce an alteration of consciousness, influence decision making and the formation of memories, which will make it more difficult to go through a perinatal grief, which in itself has its difficulties because it is considered a silenced grief.

**Conclusions:**

Quality health care in perinatal loss should focus on providing the mother with exquisite care, centred on respect for each mother’s time. This allows the establishment of a relationship that lowers fear and avoids impulses to flee, encouraging autonomy and the ability to decide, leaving the use of psychotropic drugs as a last resort for pain management.

**Disclosure of Interest:**

None Declared

